# Cytomegalovirus Diseases of the Gastrointestinal Tract in Immunocompetent Patients: A Narrative Review

**DOI:** 10.3390/v16030346

**Published:** 2024-02-23

**Authors:** Pai-Jui Yeh, Ren-Chin Wu, Chyi-Liang Chen, Cheng-Tang Chiu, Ming-Wei Lai, Chien-Chang Chen, Cheng-Hsun Chiu, Yu-Bin Pan, Wey-Ran Lin, Puo-Hsien Le

**Affiliations:** 1 Department of Pediatric Gastroenterology, Chang Gung Memorial Hospital, Linkou Branch, Taoyuan 333, Taiwan; charlie01539@hotmail.com (P.-J.Y.); a22141@cgmh.org.tw (M.-W.L.); cgj2841@cgmh.org.tw (C.-C.C.); 2 Liver Research Center, Chang Gung Memorial Hospital, Linkou Branch, Taoyuan 333, Taiwan; 3 Inflammatory Bowel Disease Center, Chang Gung Memorial Hospital, Linkou Branch, Taoyuan 333, Taiwan; renchin.wu@gmail.com (R.-C.W.); ctchiu@cgmh.org.tw (C.-T.C.); 4 Department of Pathology, Chang Gung Memorial Hospital, Linkou Branch, Taoyuan 333, Taiwan; 5 Molecular Infectious Disease Research Center, Chang Gung Memorial Hospital, Linkou Branch, Taoyuan 333, Taiwan; dinoschen@adm.cgmh.org.tw (C.-L.C.); chchiu@adm.cgmh.org.tw (C.-H.C.); 6 Department of Gastroenterology and Hepatology, Chang Gung Memorial Hospital, Linkou Branch, Taoyuan 333, Taiwan; victor.wr.lin@gmail.com; 7 Taiwan Association of the Study of Small Intestinal Disease, Taoyuan 333, Taiwan; 8 Division of Pediatric Infectious Diseases, Department of Pediatrics, Chang Gung Memorial Hospital, Linkou Branch, Taoyuan 333, Taiwan; 9 Chang Gung Microbiota Therapy Center, Chang Gung Memorial Hospital, Taoyuan 333, Taiwan; 10 Biostatistical Section, Clinical Trial Center, Chang Gung Memorial Hospital, Linkou Branch, Taoyuan 333, Taiwan; e8901145@gmail.com

**Keywords:** cytomegalovirus, immunocompetent, gastrointestinal tract

## Abstract

Cytomegalovirus (CMV) is a potential pathogen that causes gastrointestinal (GI) tract diseases regardless of host immunity. In contrast to immunocompromised individuals, immunocompetent patients lack a comprehensive overview of the gastrointestinal manifestations. This study aims to provide a comprehensive summary of the current evidence regarding presentations, diagnostics, management, risk assessment, and outcomes in immunocompetent patients with CMV GI disease. A thorough literature search of English publications up to April 2022 was conducted across electronic databases to identify relevant articles, with eligible case series selected for detailed analysis. The majority of immunocompetent patients affected by CMV GI disease are typically elderly, critically ill, or burdened with comorbidities that compromise immunity. Clinical presentations range from subtle symptoms to severe surgical conditions, including instances of mortality. Specific clinical presentations, blood test results, or endoscopic features are lacking, necessitating reliance on histopathological tests such as immunohistochemistry staining for diagnosis. While antiviral therapy may offer benefits in improving outcomes, careful individual assessment is warranted due to diverse comorbidities and potential side effects. Mortality rates vary considerably based on underlying medical conditions and therapeutic approaches. It is imperative for clinicians to maintain vigilance for CMV GI disease among high-risk groups, despite their baseline immunocompetence, in order to enhance clinical outcomes.

## 1. Introduction

Cytomegalovirus (CMV), or human herpesvirus 5 (HHV-5), is a double-stranded DNA virus with a global seroprevalence ranging from 45–100% [1,2,3,4,5]. CMV can be transmitted through saliva, blood products, breastfeeding, placenta, sexual contact, and organ transplantation [4]. With immune permissiveness and the utilization of myeloid lineage cells (CD14+ monocytes, CD34+ myeloid progenitors, dendritic cells, and megakaryocytes) as reservoirs, CMV can exhibit lifelong latency after primary infection and reactivation under an impaired immune status [2,4,6,7]. Clinical presentation varies from asymptomatic infection to tissue invasion in multiple organs [8,9]. For immunocompromised individuals, CMV has been regarded as an important opportunistic pathogen leading to mortality or morbidities, including encephalitis, pneumonitis, hepatitis, gastrointestinal (GI) tract diseases, retinitis, uveitis, nephritis, graft rejection, or severe neonatal complications in congenital infection [7,8,9,10]. 

The majority of immunocompetent patients present as asymptomatic carriers, nonspecific viral illness, or mononucleosis-like syndrome, which is usually self-limiting [4,9,10]. Despite being rare, catastrophic manifestations of CMV disease involving multiple organs in immunocompetent hosts have been reported, especially among critically ill hosts [7,8,11]. In previous studies, a small number of patients with tissue-invasive CMV had no apparent immunosuppression, risk factors, or symptoms. In some studies, nearly half of the patients were immunocompetent, which was more than “traditionally” expected for a relatively rare situation [12,13].

The GI tract is one of the most commonly targeted systems in CMV diseases [14]. CMV GI disease is defined as the presence of clinical GI symptoms, endoscopic features, and evidence of CMV in tissue via histopathology, culture, immunohistochemistry (IHC), or DNA hybridization techniques [14,15]. Reports on CMV GI disease in immunocompetent hosts in the form of case reports or case series have demonstrated a variety of clinical presentations and heterogeneous therapeutics. Only a few studies have investigated prognostic factors and performed survival analyses exclusively in immunocompetent subgroups. Several controversies remain, including the clinical significance of the detection of CMV in tissues with minimal symptoms, indications for antiviral therapy, and survival outcomes compared to immunocompromised patients [16]. Hence, this study aimed to comprehensively integrate current research on CMV GI disease through a systematic review of the literature. 

## 2. Methods

A structured literature search was performed using electronic databases (PubMed and Embase) to identify all relevant articles published before April 2022. Keywords with synonyms (in parentheses) were applied based on “cytomegalovirus (CMV)”, “immunocompetent”, and “gastrointestinal tract disease (esophagitis, gastritis, enteritis, colitis, enterocolitis, gastroenteritis, etc.)”. The primary targets were English-written case series with a full text that included no fewer than three original cases. The sources of the reported cases were checked to ensure originality and to exclude duplications. Non-English articles, conference abstracts, and those without a full text were excluded. A case series with three or more original cases was selected. Articles discussing congenital CMV disease and patients aged <1 year were excluded from the review. 

We retrieved data of interest that included disease location (esophagus, stomach, small intestine, colon, mixed [more than one location involved or unspecified]), age, sex, endoscopic feature (ulcer, polypoid lesion, inflammation), CMV status (serology, antigenemia, viremia), diagnostic methods (hematoxylin and eosin [H&E] stain, IHC, tissue polymerase chain reaction [PCR]), antiviral therapy (AVT), survival outcomes, comorbidities and risk factors (older age, ≥65 years), renal disease (acute kidney injury or chronic kidney disease), diabetes mellitus, mechanical ventilation, sepsis, inflammatory bowel disease (IBD) (diagnosed after the index CMV disease, concurrently with the CMV disease, or before the CMV disease yet receiving no immunosuppressive therapy), cancer (without chemotherapy or radiotherapy six months before the CMV disease), and special cases with unusual presentation or management. Significant findings and interpretations of outcomes were extracted as remarks. 

## 3. Results

### 3.1. Literature Search

**Figure 1:**
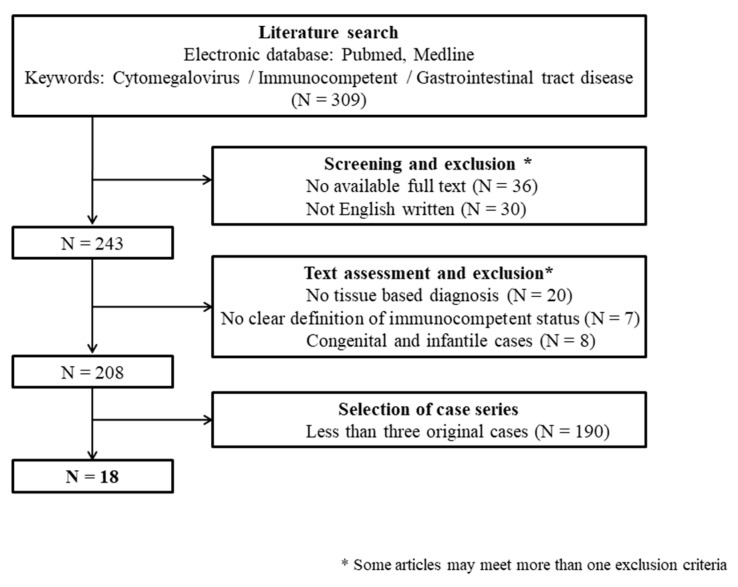
Flow chart of the literature research.

Over 200 publications were initially identified via a literature search, and 18 articles were eligible for the final analysis (Table 1) [12,13,17,18,19,20,21,22,23,24,25,26,27,28,29,30,31,32]. A flow chart of the literature selection is shown in Figure 1. 

### 3.2. Epidemiology

The prevalence of CMV GI disease in immunocompetent patients is widely different (0.3–31%) in variable subgroups [9], and accounts for 7.5–58.8% of overall CMV GI-TID (tissue invasive disease) [12,13,18,20,22,23,28,29,30]. Rafailidis et al. conducted a systematic review of severe CMV infections in immunocompetent patients, indicating that the GI tract was the primary site, involving 31% (91 out of 290) of patients [9]. Patra et al. reported an estimated CMV GI disease prevalence of three per 1000 endoscopies among patients without IBD or overt immunosuppression [20]. For critically ill immunocompetent patients, CMV reactivation and CMV GI disease developed in approximately 13.75% and 3–4% of cases, respectively [11,33]. In 78% of the enrolled studies, the mean age was >65 years, and it reached statistical significance in two cohort studies [13,30]. There is no evident sex preponderance since a wide range of male-to-female ratios have been reported. 

### 3.3. Clinical Presentations

In immunocompetent patients, the colon remains the most commonly involved segment [9]. In general, the core symptoms of immunocompetent patients are similar to those of immunocompromised patients and correlate with the involved segments. Upper GI tract diseases present with nausea, vomiting, dysphagia, odynophagia, abdominal pain, hematemesis, and tarry stool, while lower GI tract diseases present with abdominal pain, diarrhea, constipation, and bloody stool. Regardless of the involved segment, patients may have a fever and a spectrum of severities, including asymptomatic infections, nonspecific symptoms, fulminant disease, and surgical abdomen. In addition to CMV-associated toxic megacolon [34,35], CMV-related intra-abdominal abscess, fistula (rectovaginal, rectovesical), stenosis (small intestine, colon), and hollow organ perforations (stomach, small intestine, and colon) have also been reported in immunocompetent patients [36,37,38,39,40,41,42,43,44,45,46,47,48,49,50]. Compared to immunocompromised patients, more immunocompetent patients present with GI bleeding, shorter symptom onset duration, less extra-GI involvement, and more small intestinal diseases. In contrast, immunocompromised patients present with more diffuse GI tract and esophageal involvement [13,30]. Critical illnesses at diagnosis are more commonly observed in immunocompetent patients. In a study by Patra et al., all the enrolled immunocompetent patients were critically ill [20]. In addition, the percentage of immunocompetent patients needing intensive care unit (ICU) admission ranged from 21.4–39.5% and was significantly higher than that in the immunocompromised subgroup [13,27,30,31]. 

Several special manifestations that may seem atypical for common GI infections have been reported in immunocompetent patients. Patients may initially present with appendicitis, ischemic enterocolitis, or vasculitis-like features [48,51,52,53,54,55,56]. Protein-losing enteropathy, or Menetrier’s disease, can be associated, which results in edema and hypoalbuminemia [57,58,59,60,61,62,63]. CMV post-infectious motility impairment presenting with gastroparesis or constipation (segmental hypoganglionosis) has also been reported [64,65]. With relevant GI segments, preceding bacterial (*Salmonella* or *Shigella* species) infection or concurrent infection with Epstein–Barr virus (EBV), *Helicobacter pylori*, *Clostridioides difficile*, histoplasmosis, and other parasites were also noted with CMV TID [19,51,66,67,68,69,70,71,72,73,74,75,76]. A study has proposed the term “triple C disease”, which denotes “CMV *Clostridium* Colitis disease”, and it has been reported that triple C disease has not been limited to patients with IBD but also to other immunocompetent hosts [77]. While triple C disease leads to worse outcomes in IBD, the clinical significance in other immunocompetent populations still needs further study [78,79,80].

Congenital, postnatal, and infantile CMV diseases are not discussed in this review because of immature immunity. Pediatric patients are a distinctive concern, especially neonates. CMV is the leading cause of congenital and postnatal viral infections. Porta et al. conducted a literature review comprising 38 articles on neonates infected within three months of age, addressing its rare incidence and diverse associations with GI manifestations, including enteritis, colitis, necrotizing enterocolitis, perforation, appendicitis, Hirschsprung’s disease, Meckel’s diverticulum, and colonic strictures [81]. Sue et al. discussed 23 cases of invasive CMV enterocolitis among immunocompetent non-preterm children and suggested that subtle defects in the innate immune system (Toll-like receptor, nucleotide binding oligomerization domain-containing protein 2) may increase susceptibility to infection [82]. Barbati et al. summarized 89 cases of CMV-associated pediatric Menetrier’s disease, also reflecting a possible familial susceptibility to this manifestation [83]. In brief, CMV GI disease can develop in apparently immunocompetent children, varying in presentation from a self-limited condition to a surgical abdomen. 

### 3.4. Diagnosis

Clinical presentations, endoscopic features, and laboratory examinations are not specific for the diagnosis of CMV diseases of the GI tract. Histopathological examination of GI mucosal tissue is indispensable for establishing a diagnosis of CMV GI disease, and IHC staining is considered the gold standard [14,84,85,86,87,88]. H&E staining, viral culture, and nucleic acid testing (NAT) are also used for diagnosis [15,89]. H&E staining demonstrated specificity and sensitivity of 92–100% and 10–87%, respectively. The addition of IHC staining advances the sensitivity to 78–93%, and H&E staining frequently fails to detect CMV identified on extensive IHC [84]. NATs, such as PCR, yield better sensitivity and faster results; however, their expense and accessibility limit their clinical application. If both IHC and PCR were performed, the detection rate could be increased by 10–15% [90]. Although some studies were against routine IHC staining, it is still recommended for high-grade clinical or histological suspicion [91,92,93]. At the same time, IHC staining is an optimal diagnostic test in patients with IBD because AVT most benefits IHC-positive patients [84]. 

CMV blood tests (serology, antigenemia, and viremia) have poor diagnostic accuracy for CMV GI disease [4,16]. Positive IgM and viremia in CMV disease are 15–60% and 18–100%, respectively [12,26,28,30,31,89]. Even though polymorphic ulcers are the most prevalent endoscopic findings, no specific endoscopic features for CMV GI disease were found. These ulcers may complicate perforation, fistula, abscess, or stricture formation. Polypoid and tumor-like lesions were also noted [50,94,95,96,97]. There were no significant differences between the immunocompetent and immunocompromised groups, except for a study revealing a higher ratio of ulcers in the immunocompromised group (70–35%) [13,20,30].

### 3.5. Treatment

Current treatments for CMV infections include CMV immunoglobulin, ganciclovir, valganciclovir, cidofovir, foscarnet, letermovir, maribavir, and filociclovir (in development) [4,98]. Intravenous ganciclovir (acyclic guanine nucleoside analog) and oral valganciclovir (L-valyl ester, a prodrug of ganciclovir) both exhibit acceptable bioavailability and therapeutic efficacy of over 80% [14,99]. These two agents are the mainstays, whereas the rest are reserved for patients with treatment resistance or specific clinical contexts. Myelosuppression, nephrotoxicity, and electrolyte imbalance are the major adverse events during antiviral treatment [100]. Physicians should closely monitor blood cell counts, renal function, and electrolytes during AVT. 

There are no guidelines regarding the details and duration of AVT in immunocompetent patients. The therapeutic rate, regimens, and duration of AVT were heterogeneous in the available studies. In some studies, physicians tended to treat severe CMV GI diseases and found that this group of patients still had a poor prognosis due to selection bias [8,14,16]. However, adequate AVT may lead to better clinical outcomes and survival rates in immunocompetent patients [12,13,30]. We should individually evaluate the application of AVT in immunocompetent patients according to the following opinions: First, it is necessary to interpret the testing results with clues of inflammation to confirm real CMV GI diseases instead of a bystander. IHC is crucial not only for the accurate diagnosis of CMV GI disease, but AVT also benefits patients with IBD who have positive IHC staining [84]. At the same time, a positive PCR test without complementary histology or IHC findings does not always support clinically significant CMV GI disease [16]. Second, the risk of adverse events with AVT should be weighed and closely monitored throughout the treatment course. Third, AVT is indicated for critical patients with surgical conditions, comorbidities, or protracted illness, regardless of baseline immunity [8,14,101].

### 3.6. Outcomes and Prognostic Factors

The mortality rates of immunocompetent CMV GI disease ranged from 0–80% because of different subgroups and therapeutic interventions. Page et al. reported the highest mortality rate with a significantly worse survival outcome in the immunocompetent group than in the immunocompromised group, probably due to elderly status, unrecognized immunodeficiency, delayed diagnosis, and treatment [18]. Similarly, Chaemsupaphan et al. proposed a significantly higher six-month mortality rate among immunocompetent patients [30]. However, another study by Wetwittayakhlang et al. suggested no difference in in-hospital survival between the two immune statuses [13]. Furthermore, Karigane et al. reviewed 33 immunocompetent cases of CMV enteritis (including colitis, enteritis, and enterocolitis) and reported a generally favorable prognosis in view of the 24% spontaneous resolution rate and absence of CMV-related death [102]. Another study for immunocompetent CMV gastroenterocolitis by Yoon et al. also reported a spontaneous resolution rate of 38.4%, accompanied by a fair response rate (80.4%) for the rest of the AVT-treated patients [31].

With respect to prognostic factors, comprehensive analyses of immunocompetent hosts are scarce. As for CMV colitis, Ng et al. analyzed 10 cases and indicated that their overall prognosis was mainly related to comorbidities, while 75% of the deaths were irrelevant to CMV [19]. In another meta-analysis of CMV colitis in immunocompetent hosts conducted by Galiatsatos et al., the mortality rate was higher with male sex, immune modulating diseases, or a history of colectomy, albeit not statistically significant [103]. As for the whole GI tract, Yoon et al. proposed that the endoscopic type (discrete ulcer or diffuse erythema) was not associated with the clinical outcome (death or surgery), while the C-reactive protein level was the sole independent factor for poor clinical courses (surgery or in-hospital mortality) [31].

### 3.7. Risk Factors, Comorbidities, and Special Populations

Familiarity with the risk factors for CMV GI diseases helps physicians make an early diagnosis and choose the appropriate treatment to improve clinical outcomes. Ko et al. noted that steroid use and red blood cell transfusion within one month are independent risk factors for developing CMV colitis in immunocompetent patients [27]. Except for this study, the evidence of risk factors in other GI segments is limited due to the lack of comprehensive control studies, but it is worthy of further study. 

On the other hand, comorbidities worth noting include age, renal insufficiency, diabetes mellitus, and critical illness (mechanical ventilation, sepsis, shock, ICU, etc.). Patients with any of these medical conditions account for 80% of the immunocompetent patients with CMV GI disease. These conditions lead to relative immunodeficiency, even though they are immunocompetent by general definition. 

The elderly contribute in several aspects such as the general decline of organ function, malnutrition, and “immunosenescence”, which refers to changes in the immune system due to aging [28]. Involution of the thymus and reduced T-cell responses may contribute to CMV reactivation [28,104]. In addition, CMV can impair the immune defense against other pathogens by generating an expanded population of CMV-specific CD8 T cells due to reactivation, a phenomenon described as “memory inflation” [105,106]. Renal insufficiency is hypothesized to increase the risk of infection through T-cell exhaustion, immune dysregulation, and impaired clearance of pathogens [107]. In patients with end-stage renal disease, uremia leads to a reduced number and function of lymphoid cells, premature “aging” of hematopoietic stem cell composition, altered renal metabolic activities with depleted production (renin, erythropoietin, and vitamin D), and increased susceptibility to infection [108,109]. Diabetes mellitus has been shown to impair immune responses, and a meta-analysis proposed that non-autoimmune diabetes is associated with increased susceptibility to multiple types of herpesviruses [110,111,112]. During critical illness, the risk of CMV reactivation increases, possibly because of immunocompromised status, augmented stress hormone responses, and altered cytokine levels (enhanced CMV replication by tumor necrosis factor-α) [33,113]. This reactivation of CMV is frequent in critically ill, immunocompetent patients, as observed in ICU cohort studies and systematic reviews [6,11,33]. 

Patients with coexisting or successive diagnoses of IBD or cancer were classified into special subgroups. The association between IBD and CMV GI disease has been recognized for decades, especially in ulcerative colitis, although the exact mechanism remains unclear [14,89,114]. Thus, screening for CMV in patients with IBD experiencing acute flares or refractory treatments is essential. Intriguingly, a literature review by Luangsirithanya et al. described 13 cases of new-onset IBD with coexisting CMV colitis, revealing that IBD should be suspected in immunocompetent patients with CMV GI disease, especially if they have a partial response to AVT [115]. With respect to malignancy, Costa et al. reported a case of an atypical bronchopulmonary carcinoid tumor, revealed by a positron emission tomography scan arranged as a survey for possible immunodeficiency after the diagnosis of CMV duodenitis [116]. Similarly, Krajicek et al. described a patient with perforated CMV gastritis who was diagnosed with diffuse large B-cell lymphoma [47]. Ng et al. reported a case of CMV ileitis and a coexisting metastatic goblet cell carcinoid tumor [117], while Murakami et al. depicted a patient with CMV esophagitis and early esophageal cancer [118]. In three case series of CMV GI disease, a total of 12 patients were described as having successive (four as “recent”, without anti-tumor therapy) diagnoses of malignancies months after the CMV disease, involving the lung, biliary tract, pancreas, kidney, etc. [18,21,23]. In view of these observations, “occult” malignancy as an immunomodulating driver may be an important consideration for “apparently” immunocompetent hosts manifesting CMV GI disease.

## 4. Conclusions

**Figure 2:**
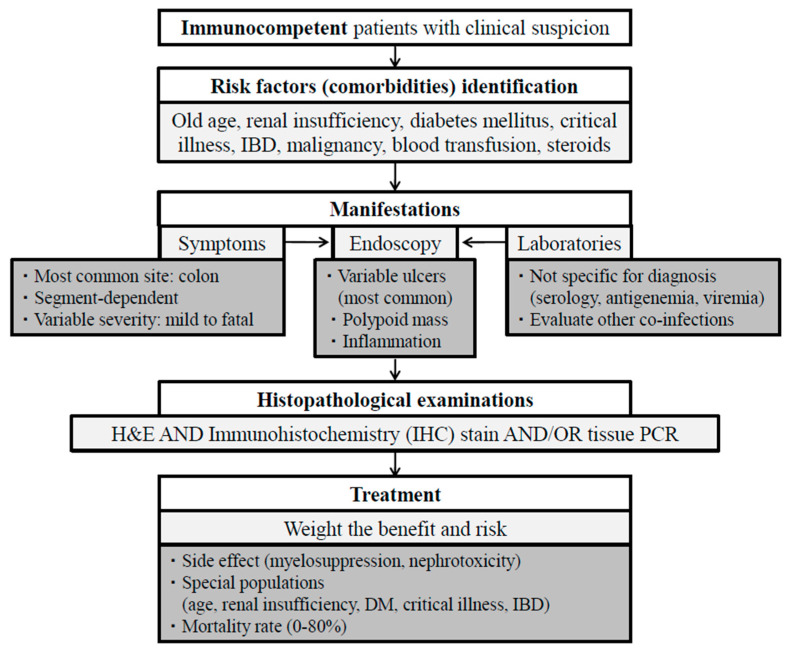
A proposed algorithm of management for immunocompetent patients with cytomegalovirus diseases of the gastrointestinal tract. H&E, hematoxylin and eosin; IBD, inflammatory bowel disease; IHC, immunohistochemistry; PCR, polymer chain reaction.

CMV GI disease in immunocompetent hosts is more common than previously thought, particularly among elderly individuals, critically ill patients, and those with underlying immunodeficiency disorders. Clinical presentations range from mild symptoms to life-threatening surgical conditions, highlighting the variability in severity. Diagnosis can be challenging as there are no specific clinical features, blood tests, or endoscopic findings. The gold standard for diagnosis remains histopathological confirmation through hematoxylin and eosin (H&E) staining and immunohistochemical (IHC) staining. AVT may offer benefits in improving outcomes, although careful individual assessment is necessary considering the presence of comorbidities and potential side effects. Mortality rates vary depending on underlying medical conditions and treatment approaches. Vigilance is crucial in recognizing CMV GI disease, especially in high-risk populations, to enhance clinical outcomes (Figure 2).

## Figures and Tables

**Table 1:** Overview of the reviewed case series.

	Author (Year)	Disease Location					Age (Years) Sex	Endoscopy			Patho.	CMV Status	AVT	Survi.	Risk Factors						
															Old	Ren	DM	MV	Sep	IBD	Can
		ES	ST	SI	CO	MX		U	P	I					Remarks						
1	Surawicz et al., 1988 [17]				3		25 (M), 37 (F), 71 (F) 1 M, 2 F	2	-	1	HE ± IHC	Infection in 2 NA in 1	0/3	3/3	1						
															Anal intercourse in two; sigmoid volvulus with colectomy; and stricture in one. All were self-limited.						
2	Page et al., 1998 [18]	1	2	2	5		NA NA	-	-	-	Biopsy proven	NA	5/10	2/10							4
															Mortality was significantly greater in the normal patient (immunocompetent) group.						
3	Ng et al., 1999 [19]				10		Median, 70; Range, 59–92; 1 M, 9 F	6	1	3	HE ± IHC	NA	3/10	6/10	7	2	3				
															Preceding events (AMI in two, *Shigella enteritis* in two). One needed surgery, and three had local complications (fistula).						
4	Patra et al., 1999 [20]		1		16		NA * NA *	6	-	11	HE	NA	NA	NA							
															All immunocompetent patients were critically ill. Immunocompromised patients had a higher ratio of ulcers (70–35%) and more atypical inclusion bodies (90–47%).						
5	Maiorana et al., 2003 [21]	1	4		6		Mean, 72; Range, 52–86; 9 M, 2 F	9	-	2	HE + IHC	IgG (+): 6/6 IgM (−): 6/6 Viremia: NA	3/11	7/11	8					2	4
															Four immunocompetent patients had malignancies of various organs (diagnosed 2–5 months later).						
6	Ng et al., 2003 [22]	1			4	1 ^a^	Mean, 74; Range, 60–81; 2 M, 4 F	4	-	2	HE ± IHC	NA	3/6	4/6	5	2	3			1	
															Total: 14 non-HIV patients, of whom only six qualified for immunocompetent status.						
7	Bonetti et al., 2011 [23]	4	11				Mean, 71; Range, 37–91; 7 M, 8 F	9	2	4	HE + IHC	IgG (+): 3/NA IgM (+): 7/NA Viremia: NA	NA	15/15	1						
															Four immunocompetent patients had malignancies of various organs (diagnosed 5–16 months later). Immunocompromised patients are more likely to have multiple segments or multiple sites of involvement. There were no differences in the frequency or localization of gastric mucosal thickenings between the two groups.						
8	Siciliano et al., 2014 [24]			2	12		Mean, 64; Range, 38–82; 6 M, 8 F	-	-	-	HE + IHC	NA	13/14	4/14	8	7	5	10	13		
															All patients developed septic or cardiogenic shock (on average, two episodes) before CMV disease. The mean in-hospital stay was 44 days, with an average of 29 days in the ICU. The in-hospital mortality rate was 71.4%. Cardiomyopathy was noted in 64.2% of patients.						
9	Chen et al., 2014 [25]	1	2		10		Mean, 68; Range, 47–77; 7 M, 6 F	8	2	3	HE ± IHC	IgM (+): 1 Antigenemia: 1	12/13	9/13	9	13	7				
															All cases had CKD; one had HSP. AVT: average of 23 ± 14 days (range, 7–42 days). Two patients died of CMV-related colonic perforation.						
10	Chan et al., 2014 [26]				4		Mean, 74; Range, 65–84; 3 M, 1 F	2	1	1	HE ± IHC	Viremia: 4	4/4	1/4	4	2	2				
															There was biopsy-proven CMV colitis in eight patients, and only four were identified as having immune status risks. Three (75%) were diagnosed via a clinician-ordered CMV stain. Stool and blood CMV-PCR were applied to diagnose “probable” cases, and stool PCR was positive in 7 of 10 tested patients.						
11	Ko et al., 2015 [27]				51		Mean ± SD, 65 ± 14; 24 M, 27 F	49	2	-	HE + IHC or PCR	IgG (+): 100% IgM (+): 8.3%	39/51	47/51	NA ^b^	16	15	11 ^c^			
															The 30-day mortality rate was 7.8% (all-cause, none related to CMV colitis directly). Risk factors for developing CMV colitis include steroid usage and RBC transfusions within one month.						
12	Bernard et al., 2015 [28]	5		1	7		Mean, 75; Range, 54–88; 5 M, 8 F	10	-	3	PCR	Reactivation: 10 Viremia: 1 (out of 4)	6/13	13/13	11	2	3			2	2
															Only one case had a positive histology finding. The mean value of CMV DNA load in GI biopsies was 3845 copies/μg total DNA. Clinical features were similar to patients diagnosed with histology methods in prior studies.						
13	Marques et al., 2017 [29]	1	2				51 (M), 86 (F), 87 (F) 1 M, 2 F	2	-	1	HE ± IHC	NA	2/3	2/3	2		1				
															In this upper GI tract cohort, 25% were immunocompetent. One had a history of ischemic stroke, and one had decompensated alcoholic liver cirrhosis and DM.						
14	Chaemsupaphan et al., 2020 [30]	4	10	15	47		Mean ± SD 73 ± 13.9 31 M, 25 F	47	6	- ^d^	HE ± IHC	Viremia: 16 (out of 27)	39/51	36/56	NA ^b^	35	20	20	NA ^e^		
															AVT is an independent protective factor *. Predictors of six-month mortality: age, inpatient status, ICU *. Patients in the immunocompetent group were older, had more ICU needs at diagnosis, had more viremia-negative cases, were less treated, and had higher six-month mortality (39% vs. 22%).						
15	Wetwittayakhlang et al., 2021 [13]	5	4	17	59	4	Median, 70; IQR, 63–79; 51 M, 38 F	58	3	28	HE ± IHC	NA	80/89	64/89	59	56	12	34	30	4	
															AVT > 14 days was a protective factor for survival (Peto-Peto test, *p* < 0.001) *. The immunocompetent group was older, had more GI bleeding, shorter symptom-onset duration, and had more involvement in SI and less in ES/MX. There was no difference in in-hospital mortality regarding immunity.						
16	Yoon et al., 2021 [31]				60	26 ^f^	Median, 68; IQR, 60–74; 53 M, 33 F	55	-	31	HE ± IHC ± PCR	Viremia: 25 (out of 46)	51/86	78/86	NA ^b^	15	34	34	36		
															Sixty-eight cases (79.1%) had comorbidities. Endoscopic features were not associated with clinical outcomes. CRP is an independent risk factor for surgery and in-hospital mortality.						
17	Verma et al., 2021 [32]				4		45 (M), 52 (M), 55 (F), 65 (F) 2 M, 2 F	4	-	-	HE + IHC ± PCR	NA	3/4	3/4	1		2				
															One case had a history of coronavirus disease 2019. One was positive for tissue CMV PCR. One patient died of a myocardial infarction before receiving AVT.						
18	Yeh et al., 2022 [12]	12	32	9	127		Mean ± SD, 65.3 ± 17.6; 105 M, 75 F	148	22	10	HE + IHC	IgG (+): 97.2% IgM (+): 16.9% Antigenemia: 42.9% Viremia: 65.9%	99/180	152/180	108	79	75	42		28	20
															Immunocompetent patients receiving Combo therapy had the best survival curve. Combo AVT (oral plus intravenous) ≥ 14 days resulted in better outcomes for both immunocompromised and immunocompetent patients.						

* Not exclusively for the immunocompetent subgroup. ^a^, duodenum plus colon; ^b^, no definite case number; ^c^, presumed numbers according to the ICU requirement; ^d^, unable to identify pure inflammation based on the data; ^e^, no definite case number (bacteremia in 4, systemic inflammatory response syndrome in 29, shock in 19); ^f^, upper GI in 19, SB in 7. Abbreviations: AMI, acute myocardial infarction; AVT, antiviral therapy; Can, cancer; CKD, chronic kidney disease; CMV, cytomegalovirus; CO, colon; Combo, combination (oral and intravenous form); CRP, C-reactive protein; DM, diabetes mellitus; ES, esophagus; F, female; GI, gastrointestinal; HE, hematoxylin and eosin stain; HSP, Henoch-Schönlein purpura; I, inflammation; IBD, inflammatory bowel disease; ICU, intensive care unit; IHC, immunohistochemical staining; IQR, interquartile range; M, male; MV, mechanical ventilation; MX, mixed segments; NA, not available; P, polypoid lesion; Patho., pathology; PCR, polymerase chain reaction; RBC, red blood cell; Ren, renal disease; SD, standard deviation; Sep, sepsis; SI, small intestine; ST, stomach; Survi., survival; U, ulcer.

## References

[1] Herbein G. (2022). Tumors and Cytomegalovirus: An Intimate Interplay. Viruses.

[2] You D.M., Johnson M.D. (2012). Cytomegalovirus infection and the gastrointestinal tract. Curr. Gastroenterol. Rep..

[3] Cannon M.J., Schmid D.S., Hyde T.B. (2010). Review of cytomegalovirus seroprevalence and demographic characteristics associated with infection. Rev. Med. Virol..

[4] Gandhi M.K., Khanna R. (2004). Human cytomegalovirus: Clinical aspects, immune regulation, and emerging treatments. Lancet Infect. Dis..

[5] Staras S.A., Dollard S.C., Radford K.W., Flanders W.D., Pass R.F., Cannon M.J. (2006). Seroprevalence of cytomegalovirus infection in the United States, 1988–1994. Clin. Infect. Dis..

[6] Limaye A.P., Kirby K.A., Rubenfeld G.D., Leisenring W.M., Bulger E.M., Neff M.J., Gibran N.S., Huang M.L., Santo Hayes T.K., Corey L. (2008). Cytomegalovirus reactivation in critically ill immunocompetent patients. JAMA.

[7] Nakase H., Herfarth H. (2016). Cytomegalovirus Colitis, Cytomegalovirus Hepatitis and Systemic Cytomegalovirus Infection: Common Features and Differences. Inflamm. Intest. Dis..

[8] Lancini D., Faddy H.M., Flower R., Hogan C. (2014). Cytomegalovirus disease in immunocompetent adults. Med. J. Aust..

[9] Rafailidis P.I., Mourtzoukou E.G., Varbobitis I.C., Falagas M.E. (2008). Severe cytomegalovirus infection in apparently immunocompetent patients: A systematic review. Virol. J..

[10] Eddleston M., Peacock S., Juniper M., Warrell D.A. (1997). Severe cytomegalovirus infection in immunocompetent patients. Clin. Infect. Dis..

[11] Osawa R., Singh N. (2009). Cytomegalovirus infection in critically ill patients: A systematic review. Crit. Care.

[12] Yeh P.J., Wu R.C., Chiu C.T., Lai M.W., Chen C.M., Pan Y.B., Su M.Y., Kuo C.J., Lin W.R., Le P.H. (2022). Cytomegalovirus Diseases of the Gastrointestinal Tract. Viruses.

[13] Wetwittayakhlang P., Rujeerapaiboon N., Wetwittayakhlung P., Sripongpun P., Pruphetkaew N., Jandee S., Chamroonkul N., Piratvisuth T. (2021). Clinical Features, Endoscopic Findings, and Predictive Factors for Mortality in Tissue-Invasive Gastrointestinal Cytomegalovirus Disease between Immunocompetent and Immunocompromised Patients. Gastroenterol. Res. Pract..

[14] Fakhreddine A.Y., Frenette C.T., Konijeti G.G. (2019). A Practical Review of Cytomegalovirus in Gastroenterology and Hepatology. Gastroenterol. Res. Pract..

[15] Ljungman P., Boeckh M., Hirsch H.H., Josephson F., Lundgren J., Nichols G., Pikis A., Razonable R.R., Miller V., Griffiths P.D. (2016). Definitions of Cytomegalovirus Infection and Disease in Transplant Patients for Use in Clinical Trials. Clin. Infect. Dis..

[16] Goodman A.L., Murray C.D., Watkins J., Griffiths P.D., Webster D.P. (2015). CMV in the gut: A critical review of CMV detection in the immunocompetent host with colitis. Eur. J. Clin. Microbiol. Infect. Dis..

[17] Surawicz C.M., Myerson D. (1988). Self-limited cytomegalovirus colitis in immunocompetent individuals. Gastroenterology.

[18] Page M.J., Dreese J.C., Poritz L.S., Koltun W.A. (1998). Cytomegalovirus enteritis: A highly lethal condition requiring early detection and intervention. Dis. Colon. Rectum.

[19] Ng F.H., Chau T.N., Cheung T.C., Kng C., Wong S.Y., Ng W.F., Lee K.C., Chan E., Lai S.T., Yuen W.C. (1999). Cytomegalovirus colitis in individuals without apparent cause of immunodeficiency. Dig. Dis. Sci..

[20] Patra S., Samal S.C., Chacko A., Mathan V.I., Mathan M.M. (1999). Cytomegalovirus infection of the human gastrointestinal tract. J. Gastroenterol. Hepatol..

[21] Maiorana A., Baccarini P., Foroni M., Bellini N., Giusti F. (2003). Human cytomegalovirus infection of the gastrointestinal tract in apparently immunocompetent patients. Hum. Pathol..

[22] Ng K.L., Ho J., Ng H.S., Luman W. (2003). Gastrointestinal Cytomegalovirus infection in non-human immunodeficiency virus infected patients. Med. J. Malays..

[23] Reggiani Bonetti L., Losi L., Di Gregorio C., Bertani A., Merighi A., Bettelli S., Scuri M., Maiorana A. (2011). Cytomegalovirus infection of the upper gastrointestinal tract: A clinical and pathological study of 30 cases. Scand. J. Gastroenterol..

[24] Siciliano R.F., Castelli J.B., Randi B.A., Vieira R.D., Strabelli T.M. (2014). Cytomegalovirus colitis in immunocompetent critically ill patients. Int. J. Infect. Dis..

[25] Chen Y.-M., Hung Y.-P., Huang C.-F., Lee N.-Y., Chen C.-Y., Sung J.-M., Chang C.-M., Chen P.-L., Lee C.-C., Wu Y.-H. (2014). Cytomegalovirus disease in nonimmunocompromised, human immunodeficiency virus-negative adults with chronic kidney disease. J. Microbiol. Immunol. Infect..

[26] Chan K.-S., Yang C.-C., Chen C.-M., Yang H.-H., Lee C.-C., Chuang Y.-C., Yu W.-L. (2014). Cytomegalovirus colitis in intensive care unit patients: Difficulties in clinical diagnosis. J. Crit. Care.

[27] Ko J.H., Peck K.R., Lee W.J., Lee J.Y., Cho S.Y., Ha Y.E., Kang C.I., Chung D.R., Kim Y.H., Lee N.Y. (2015). Clinical presentation and risk factors for cytomegalovirus colitis in immunocompetent adult patients. Clin. Infect. Dis..

[28] Bernard S., Germi R., Lupo J., Laverrière M.H., Masse V., Morand P., Gavazzi G. (2015). Symptomatic cytomegalovirus gastrointestinal infection with positive quantitative real-time PCR findings in apparently immunocompetent patients: A case series. Clin. Microbiol. Infect..

[29] Marques S., Carmo J., Pinto D., Bispo M., Ramos S., Chagas C. (2017). Cytomegalovirus Disease of the Upper Gastrointestinal Tract: A 10-Year Retrospective Study. GE Port. J. Gastroenterol..

[30] Chaemsupaphan T., Limsrivilai J., Thongdee C., Sudcharoen A., Pongpaibul A., Pausawasdi N., Charatcharoenwitthaya P. (2020). Patient characteristics, clinical manifestations, prognosis, and factors associated with gastrointestinal cytomegalovirus infection in immunocompetent patients. BMC Gastroenterol..

[31] Yoon J., Lee J., Kim D.S., Lee J.W., Hong S.W., Hwang H.W., Hwang S.W., Park S.H., Yang D.H., Ye B.D. (2021). Endoscopic features and clinical outcomes of cytomegalovirus gastroenterocolitis in immunocompetent patients. Sci. Rep..

[32] Verma A., Girish M.I., Dahale A.S., Dalal A., Sachdeva S. (2021). CMV Colitis in Immunocompetent Patients—A Case Series. J. Dig. Endosc..

[33] Frantzeskaki F.G., Karampi E.S., Kottaridi C., Alepaki M., Routsi C., Tzanela M., Vassiliadi D.A., Douka E., Tsaousi S., Gennimata V. (2015). Cytomegalovirus reactivation in a general, nonimmunosuppressed intensive care unit population: Incidence, risk factors, associations with organ dysfunction, and inflammatory biomarkers. J. Crit. Care.

[34] Lin Y.H., Yeh C.J., Chen Y.J., Chang M.C., Su I.H., Cheng H.T. (2010). Recurrent cytomegalovirus colitis with megacolon in an immunocompetent elderly man. J. Med. Virol..

[35] Cho J.H., Choi J.H. (2020). Cytomegalovirus ileo-pancolitis presenting as toxic megacolon in an immunocompetent patient: A case report. World J. Clin. Cases.

[36] Buckner F.S., Pomeroy C. (1993). Cytomegalovirus disease of the gastrointestinal tract in patients without AIDS. Clin. Infect. Dis..

[37] Petrogiannopoulos C.L., Kalogeropoulos S.G., Dandakis D.C., Hartzoulakis G.A., Karahalios G.N., Flevaris C.P., Zacharof A.K. (2004). Cytomegalovirus enteritis in an immunocompetent host. Chemotherapy.

[38] Yun E.J., Han J.K., Choi B.I. (1999). Cytomegalovirus proctitis in a diabetic. Abdom. Imaging.

[39] Ashida Y., Kashihara T., Masuda E., Doi Y., Murayama Y., Okuno M., Kimura F. (2006). A case of cytomegalovirus proctitis associated with both rectovaginal and rectovesical fistulae. Gastroenterol. Endosc..

[40] Alam I., Shanoon D., Alhamdani A., Boyd A., Griffiths A.P., Baxter J.N. (2007). Severe proctitis, perforation, and fatal rectal bleeding secondary to cytomegalovirus in an immunocompetent patient: Report of a case. Surg. Today.

[41] Cha J.M., Lee J.I., Choe J.W., Joo K.R., Jung S.W., Shin H.P., Choi S.I. (2010). Cytomegalovirus enteritis causing ileal perforation in an elderly immunocompetent individual. Yonsei Med. J..

[42] Tejedor Cerdeña M.A., Velasco Guardado A., Fernández Prodomingo A., Concepción Piñero Pérez M.C., Calderón R., Prieto Bermejo A.B., Sánchez Garrido A., Martínez Moreno J., Geijo Martínez F., Blanco Múñez O.J. (2011). Cytomegalovirus ileitis in an immunocompetent patient. Rev. Esp. Enferm. Dig..

[43] Dinesh B.V., Selvaraju K., Kumar S., Thota S. (2013). Cytomegalovirus-induced colonic stricture presenting as acute intestinal obstruction in an immunocompetent adult. BMJ Case Rep..

[44] Park J.-W., Kim K., Yoon S.-W., Kim D. (2014). Cytomegalovirus Enteritis in an Immunocompetent Patient Causing Small Bowel Obstruction and Superior Mesenteric Artery Thrombosis: A Case Report. J. Korean Soc. Radiol..

[45] Hasegawa T., Aomatsu K., Nakamura M., Aomatsu N., Aomatsu K. (2015). Cytomegalovirus colitis followed by ischemic colitis in a non-immunocompromised adult: A case report. World J. Gastroenterol..

[46] Chidlovskii E., Deroux A., Bernard S., Couturier P. (2016). Cytomegalovirus colitis mimicking rectal carcinoma in an immunocompetent elderly woman. BMJ Case Rep..

[47] Krajicek E., Shivashankar R., Hansel S. (2017). Cytomegalovirus and the Seemingly Immunocompetent Host: A Case of a Perforating Gastric Ulcer. ACG Case Rep. J..

[48] D’Cruz R.T., Lau C.C., Thamboo T.P. (2018). Severe ischemic cytomegalovirus proctocolitis with multiple perforation. Arch. Virol..

[49] Stilwell K.T., Estes J., Kurtz M.T., Francis J.M., Lynch D.T., Patel A.A. (2019). CMV Ileitis: To Treat or Not to Treat? Implications of Initiating Biologic Therapy for Concurrent Crohn’s Disease. Case Rep. Gastrointest. Med..

[50] Santacruz C.C., Carlin P.S., Rancano R.S., Medina L.O., Miguel J.C. (2019). Segmental cytomegalovirus colitis mimicking sigmoid tumor in an immunocompetent patient. Turk. J. Gastroenterol..

[51] Kanafani Z., Sharara A., Shabb N., Kanj S. (2004). Cytomegalovirus Appendicitis Following Acute Epstein-Barr Virus Infection in an Immunocompetent Patient. Scand. J. Infect. Dis..

[52] Pasticci M.B., Corsi S., Spigarelli F., Correnti S., Francisci D., Castronari R., Baldin P., Prosperini A., Baldelli F., Cenci E. (2014). Acute appendicitis due to Cytomegalovirus in an apparently immunocompetent patient: A case report. J. Med. Case Rep..

[53] Canterino J.E., McCormack M., Gurung A., Passarelli J., Landry M.L., Golden M. (2016). Cytomegalovirus appendicitis in an immunocompetent host. J. Clin. Virol..

[54] Morunglav M., Theate I., Bertin G., Hantson P. (2010). CMV enteritis causing massive intestinal hemorrhage in an elderly patient. Case Rep. Med..

[55] D’Alessandro M., Buoncompagni A., Minoia F., Coccia M.C., Martini A., Picco P. (2014). Cytomegalovirus-related necrotising vasculitis mimicking Henoch-Schönlein syndrome. Clin. Exp. Rheumatol..

[56] Puerta A., Priego P., Galindo J. (2017). Cytomegalovirus: Associated ischemic colitis in an immunocompetent patient. Rev. Esp. Enferm. Dig..

[57] Nakase H., Itani T., Mimura J., Takeuchi R., Kawasaki T., Komori H., Hashimoto K., Chiba T. (1998). Transient protein-losing enteropathy associated with cytomegalovirus infection in a noncompromised host: A case report. Am. J. Gastroenterol..

[58] Suter W.R., Neuweiler J., Borovicka J., Binek J., Fantin A.C., Meyenberger C. (2000). Cytomegalovirus-Induced Transient Protein-Losing Hypertrophic Gastropathy in an Immunocompetent Adult. Digestion.

[59] Setakhr V., Muller G., Hoang P., Lambert A.S., Geubel A. (2007). Cytomegalovirus-associated protein losing gastropathy in an immunocompetent adult: A case report. Acta Gastroenterol. Belg..

[60] Canan O., Ozçay F., Bilezikçi B. (2008). Ménétrier’s disease and severe gastric ulcers associated with cytomegalovirus infection in an immunocompetent child: A case report. Turk. J. Pediatr..

[61] Lalazar G., Doviner V., Ben-Chetrit E. (2014). Unfolding the Diagnosis. N. Engl. J. Med..

[62] Perrineau S., Cazals-Hatem D., Zarrouk V., Fantin B., de Lastours V. (2017). Cytomegalovirus-associated protein-losing enteropathy in a healthy man. Med. Mal. Infect..

[63] Ochiai Y., Hoteya S., Kono K., Takazawa Y., Matsui A., Kikuchi D. (2021). Cytomegalovirus ileitis with protein-losing enteropathy in an immunocompetent adult. Clin. J. Gastroenterol..

[64] Solito S., Marino M., Pevere S., Scardino G., MacChini F., Vadalà Di Prampero S.F., Zilli M. (2018). Gastroparesis and pancytopenia due to cytomegalovirus infection in an immunocompetent host. Dig. Liver Dis..

[65] Kim B.S., Park S.Y., Kim D.H., Kim N.I., Yoon J.H., Ju J.K., Park C.H., Kim H.S., Choi S.K. (2021). Cytomegalovirus colitis induced segmental colonic hypoganglionosis in an immunocompetent patient: A case report. World J. Clin. Cases.

[66] Kurtz M., Morgan M. (2012). Concomitant Clostridium difficile colitis and cytomegalovirus colitis in an immunocompetent elderly female. BMJ Case Rep..

[67] Chen S., Lalazar G., Barak O., Adar T., Doviner V., Mizrahi M. (2012). Protein-Loosing Entropathy Induced by Unique Combination of CMV and HP in an Immunocompetent Patient. Case Rep. Med..

[68] Dumitru I.M., Dumitru E., Resul G., Curtali L., Paris S., Rugina S. (2014). Concomitant CMV and Clostridium difficile colitis in an immunocompetent patient treated with Ganciclovir and fecal transplantation. J. Gastrointestin Liver Dis..

[69] Claeys M., Cool M., Lambrecht G.L., Hertveldt K., Alliet G., Deboever G. (2015). CMV gastritis in the immunocompetent host. Acta Gastroenterol. Belg..

[70] Chen P.-H., Lu I.T., Lee B.-J., Wang C.-Y., Lee C.-K. (2015). Age can be a Problem: Clostridium difficile and Cytomegalovirus Colitis Coinfection in an Immunocompetent 90-year-old Patient. Int. J. Gerontol..

[71] Harano Y., Kotajima L., Arioka H. (2015). Case of cytomegalovirus colitis in an immunocompetent patient: A rare cause of abdominal pain and diarrhea in the elderly. Int. J. Gen. Med..

[72] Nasa M., Patel N., Lipi L., Sud R. (2017). Gastrointestinal Histoplasmosis and CMV Co-Infection in an Immunocompetent Host. J. Assoc. Physicians India.

[73] Yamamoto S., Sakai Y. (2019). Acute gastritis caused by concurrent infection with Epstein-Barr virus and cytomegalovirus in an immunocompetent adult. Clin. J. Gastroenterol..

[74] Seminari E., Fronti E., Contardi G., Broglia F., Scevola D., Fiorina L., Baldanti F. (2012). Colitis in an elderly immunocompetent patient. J. Clin. Virol..

[75] Bernardes C., Quaresma F., Capela T., Saiote J. (2018). Severe Cytomegalovirus ileitis preceded by acute bacterial enteritis in an immunocompetent patient. Acta Gastroenterol. Belg..

[76] Chan K.S., Lee W.Y., Yu W.L. (2016). Coexisting cytomegalovirus infection in immunocompetent patients with Clostridium difficile colitis. J. Microbiol. Immunol. Infect..

[77] Alkhatib A.A., Tietze C.C., Peterson K.A., Go M.F. (2009). Cytomegalovirus clostridium colitis disease in an immunocompetent patient. South. Med. J..

[78] McCurdy J., Enders F., Khanna S., Bruining D., Jones A., Killian J., Tariq R., Smyrk T., Loftus E. (2016). Increased Rates of Clostridium difficile Infection and Poor Outcomes in Patients with IBD with Cytomegalovirus. Inflamm. Bowel Dis..

[79] Li Y., Xu H., Xu T., Xiao M., Tang H., Wu D., Tan B., Li J., Yang H., Lv H. (2018). Case–Control Study of Inflammatory Bowel Disease Patients with and without Clostridium difficile Infection and Poor Outcomes in Patients Coinfected with C. difficile and Cytomegalovirus. Dig. Dis. Sci..

[80] Xu H., Tang H., Xu T., Xiao M., Li J., Tan B., Yang H., Lv H., Li Y., Qian J. (2019). Retrospective analysis of Clostridium difficile infection in patients with ulcerative colitis in a tertiary hospital in China. BMC Gastroenterol..

[81] Porta A., Avanzini A., Bellini M., Crossignani R.M., Fiocchi S., Martinelli S., Parola L. (2016). Neonatal gastrointestinal involvement and congenital cytomegalovirus. Pediatr. Med. Chir..

[82] Sue P.K., Salazar-Austin N.M., McDonald O.G., Rishi A., Cornish T.C., Arav-Boger R. (2016). Cytomegalovirus Enterocolitis in Immunocompetent Young Children: A Report of Two Cases and Review of the Literature. Pediatr. Infect. Dis. J..

[83] Barbati F., Marrani E., Indolfi G., Lionetti P., Trapani S. (2021). Menetrier disease and Cytomegalovirus infection in paediatric age: Report of three cases and a review of the literature. Eur. J. Pediatr..

[84] Johnson J., Affolter K., Boynton K., Chen X., Valentine J., Peterson K. (2018). CMV Disease in IBD: Comparison of Diagnostic Tests and Correlation with Disease Outcome. Inflamm. Bowel Dis..

[85] Yerushalmy-Feler A., Padlipsky J., Cohen S. (2019). Diagnosis and Management of CMV Colitis. Curr. Infect. Dis. Rep..

[86] Hazır-Konya H., Avkan-Oğuz V., Akpınar H., Sağol Ö., Sayıner A. (2021). Investigation of Cytomegalovirus in Intestinal Tissue in a Country With High CMV Seroprevalence. Turk. J. Gastroenterol..

[87] Burston J., van Hal S., Dubedat S., Lee A. (2017). Inclusions or bystanders? CMV PCR sensitivity and specificity in tissue samples. J. Clin. Virol..

[88] Yan Z., Wang L., Dennis J., Doern C., Baker J., Park J.Y. (2014). Clinical significance of isolated cytomegalovirus-infected gastrointestinal cells. Int. J. Surg. Pathol..

[89] Mourad F.H., Hashash J.G., Kariyawasam V.C., Leong R.W. (2020). Ulcerative Colitis and Cytomegalovirus Infection: From A to Z. J. Crohn’s Colitis.

[90] Mills A.M., Guo F.P., Copland A.P., Pai R.K., Pinsky B.A. (2013). A comparison of CMV detection in gastrointestinal mucosal biopsies using immunohistochemistry and PCR performed on formalin-fixed, paraffin-embedded tissue. Am. J. Surg. Pathol..

[91] Solomon I.H., Hornick J.L., Laga A.C. (2017). Immunohistochemistry Is Rarely Justified for the Diagnosis of Viral Infections. Am. J. Clin. Pathol..

[92] Guo L., DeRoche T.C., Salih Z.T., Qasem S.A. (2018). Routine Hematoxylin and Eosin Stain Is Specific for the Diagnosis of Cytomegalovirus Infection in Gastrointestinal Biopsy Specimens. Int. J. Surg. Pathol..

[93] Juric-Sekhar G., Upton M.P., Swanson P.E., Westerhoff M. (2017). Cytomegalovirus (CMV) in gastrointestinal mucosal biopsies: Should a pathologist perform CMV immunohistochemistry if the clinician requests it?. Hum. Pathol..

[94] Kawasaki S., Osawa S., Sugimoto K., Uotani T., Nishino M., Yamada T., Sugimoto M., Furuta T., Ikuma M. (2010). Cecal vanishing tumor associated with cytomegalovirus infection in an immunocompetent elderly adult. World J. Gastrointest. Oncol..

[95] Agaimy A., Mudter J., Märkl B., Chetty R. (2011). Cytomegalovirus infection presenting as isolated inflammatory polyps of the gastrointestinal tract. Pathology.

[96] Xiong X., Liu F., Zhao W., Ji X., Chen W., Zou H., Li F. (2019). Cytomegalovirus infective gastritis in an immunocompetent host misdiagnosed as malignancy on upper gastrointestinal endoscopy: A case report and review of literature. Hum. Pathol..

[97] Vegunta A.S., Dasar S.K., Joshi S.K., Rao R.V. (2015). Spontaneous Partial Vanishing Cytomegalovirus Pseudotumour of Colon in an Immunocompetent Patient. J. Clin. Diagn. Res..

[98] Hakki M. (2020). Moving Past Ganciclovir and Foscarnet: Advances in CMV Therapy. Curr. Hematol. Malig. Rep..

[99] Vaziri S., Pezhman Z., Sayyad B., Mansouri F., Janbakhsh A., Afsharian M., Najafi F. (2014). Efficacy of valganciclovir and ganciclovir for cytomegalovirus disease in solid organ transplants: A meta-analysis. J. Res. Med. Sci..

[100] Upadhyayula S., Michaels M.G. (2013). Ganciclovir, Foscarnet, and Cidofovir: Antiviral Drugs Not Just for Cytomegalovirus. J. Pediatr. Infect. Dis. Soc..

[101] Klauber E., Briski L.E., Khatib R. (1998). Cytomegalovirus colitis in the immunocompetent host: An overview. Scand. J. Infect. Dis..

[102] Karigane D., Takaya S., Seki Y., Mastumoto Y., Onose A., Kosakai A., Sugaya N., Mori T. (2014). Cytomegalovirus enteritis in immunocompetent subjects: A case report and review of the literature. J. Infect. Chemother..

[103] Galiatsatos P., Shrier I., Lamoureux E., Szilagyi A. (2005). Meta-analysis of outcome of cytomegalovirus colitis in immunocompetent hosts. Dig. Dis. Sci..

[104] Naylor K., Li G., Vallejo A.N., Lee W.W., Koetz K., Bryl E., Witkowski J., Fulbright J., Weyand C.M., Goronzy J.J. (2005). The influence of age on T cell generation and TCR diversity. J. Immunol..

[105] Nikolich-Žugich J., van Lier R.A.W. (2017). Cytomegalovirus (CMV) research in immune senescence comes of age: Overview of the 6th International Workshop on CMV and Immunosenescence. Geroscience.

[106] Solana R., Tarazona R., Aiello A.E., Akbar A.N., Appay V., Beswick M., Bosch J.A., Campos C., Cantisán S., Cicin-Sain L. (2012). CMV and Immunosenescence: From basics to clinics. Immun. Ageing.

[107] Hartzell S., Bin S., Cantarelli C., Haverly M., Manrique J., Angeletti A., Manna G., Murphy B., Zhang W., Levitsky J. (2020). Kidney Failure Associates With T Cell Exhaustion and Imbalanced Follicular Helper T Cells. Front. Immunol..

[108] Betjes M.G.H. (2013). Immune cell dysfunction and inflammation in end-stage renal disease. Nat. Rev. Nephrol..

[109] Cohen G. (2020). Immune Dysfunction in Uremia 2020. Toxins.

[110] Janssen A.W.M., Stienstra R., Jaeger M., van Gool A.J., Joosten L.A.B., Netea M.G., Riksen N.P., Tack C.J. (2021). Understanding the increased risk of infections in diabetes: Innate and adaptive immune responses in type 1 diabetes. Metabolism.

[111] Carey I.M., Critchley J.A., DeWilde S., Harris T., Hosking F.J., Cook D.G. (2018). Risk of Infection in Type 1 and Type 2 Diabetes Compared With the General Population: A Matched Cohort Study. Diabetes Care.

[112] Lontchi-Yimagou E., Feutseu C., Kenmoe S., Djomkam Zune A.L., Kinyuy Ekali S.F., Nguewa J.L., Choukem S.P., Mbanya J.C., Gautier J.F., Sobngwi E. (2021). Non-autoimmune diabetes mellitus and the risk of virus infections: A systematic review and meta-analysis of case-control and cohort studies. Sci. Rep..

[113] Al-Omari A., Aljamaan F., Alhazzani W., Salih S., Arabi Y. (2016). Cytomegalovirus infection in immunocompetent critically ill adults: Literature review. Ann. Intensive Care.

[114] Sager K., Alam S., Bond A., Chinnappan L., Probert C.S. (2015). Review article: Cytomegalovirus and inflammatory bowel disease. Aliment. Pharmacol. Ther..

[115] Luangsirithanya P., Treewaree S., Pongpaibul A., Pausawasdi N., Limsrivilai J. (2021). Cytomegalovirus enterocolitis with subsequent diagnosis of coexisting new-onset inflammatory bowel disease: Two case reports and review of the literature. Medicine.

[116] Costa D., Fernandes D., Furtado A., Santa Cruz A. (2017). Cytomegalovirus duodenitis in immunocompetent patients: What else should we look for?. BMJ Case Rep..

[117] Ng S.C., Noursadeghi M., von Herbay A., Vaizey C., Pitcher M.C., Flanagan K.L. (2007). Cytomegalovirus ileitis associated with goblet cell carcinoid tumour of the appendix. J. Infect..

[118] Murakami D., Harada H., Yamato M., Amano Y. (2021). Cytomegalovirus-associated esophagitis on early esophageal cancer in immunocompetent host: A case report. Gut Pathog..

